# Immunometabolism in cancer: basic mechanisms and new targeting strategy

**DOI:** 10.1038/s41420-024-02006-2

**Published:** 2024-05-16

**Authors:** Ranran Su, Yingying Shao, Manru Huang, Donghui Liu, Haiyang Yu, Yuling Qiu

**Affiliations:** 1 State Key Laboratory of Component‐Based Chinese Medicine, Tianjin University of Traditional Chinese Medicine, Tianjin, China; 2 Key Laboratory of Pharmacology of Traditional Chinese Medical Formulae, Ministry of Education, Tianjin University of Traditional Chinese Medicine, Tianjin, China; 3 Haihe Laboratory of Modern Chinese Medicine, Tianjin, China; 4 School of Pharmacy, Tianjin Medical University, Tianjin, China

**Keywords:** Cancer microenvironment, Phosphoinositol signalling, Cancer metabolism

## Abstract

Maturing immunometabolic research empowers immune regulation novel approaches. Progressive metabolic adaptation of tumor cells permits a thriving tumor microenvironment (TME) in which immune cells always lose the initial killing capacity, which remains an unsolved dilemma even with the development of immune checkpoint therapies. In recent years, many studies on tumor immunometabolism have been reported. The development of immunometabolism may facilitate anti-tumor immunotherapy from the recurrent crosstalk between metabolism and immunity. Here, we discuss clinical studies of the core signaling pathways of immunometabolism and their inhibitors or agonists, as well as the specific functions of these pathways in regulating immunity and metabolism, and discuss some of the identified immunometabolic checkpoints. Understanding the comprehensive advances in immunometabolism helps to revise the status quo of cancer treatment.

**Figure:**
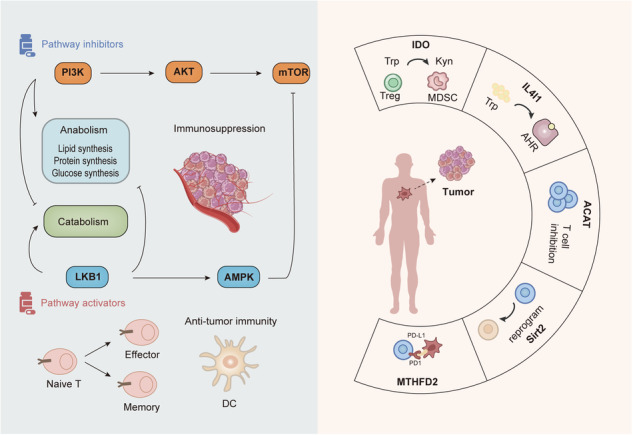
**An overview of the new landscape of immunometabolism**. The PI3K pathway promotes anabolism and inhibits catabolism. The LKB1 pathway inhibits anabolism and promotes catabolism. Overactivation of PI3K/AKT/mTOR pathway and IDO, IL4I1, ACAT, Sirt2, and MTHFD2 promote immunosuppression of TME formation, as evidenced by increased Treg and decreased T-cell proliferation. The LKBI-AMPK pathway promotes the differentiation of naive T cells to effector T cells and memory T cells and promotes anti-tumor immunity in DCs.

**An overview of the new landscape of immunometabolism**. The PI3K pathway promotes anabolism and inhibits catabolism. The LKB1 pathway inhibits anabolism and promotes catabolism. Overactivation of PI3K/AKT/mTOR pathway and IDO, IL4I1, ACAT, Sirt2, and MTHFD2 promote immunosuppression of TME formation, as evidenced by increased Treg and decreased T-cell proliferation. The LKBI-AMPK pathway promotes the differentiation of naive T cells to effector T cells and memory T cells and promotes anti-tumor immunity in DCs.

## Facts

- Metabolic vulnerability of immune cells in the TME.
- Targeting the metabolic pathways of immune cells is an effective way to modulate the immune response.
- The development of immunometabolism may facilitate anti-tumor immunotherapy from the crosstalk between metabolism and immunity.
- PI3K/AKT/mTOR and LKB1-AMPK are core signaling pathways in immunometabolism.
- Immunometabolic checkpoints have great potential.

## Open questions

- How to reverse the metabolic vulnerability of immune cells in TME?
- How can we use immunometabolic modulation to inhibit cancer cell growth?
- How do the PI3K/AKT/mTOR and LKB1-AMPK pathways regulate immunometabolism?
- What are the potential findings of immunometabolic checkpoints?

## Introduction

Cancer is a serious global health problem. Although traditional therapies have prolonged many patients’ lives, the presence of stronger side effects has prompted researchers to work toward finding a method that can specifically destroy tumor cells without affecting normal cells [1, 2]. Understanding the multi-effector molecules of immunometabolism will confer more clinical research significance on tumor therapy [3]. Alternative metabolic pathways are regulated by tumor cells to adapt to specific nutrients. Cancer cells constantly exhibit metabolic reprogramming during proliferation which mediates immune escape as well as drug resistance [4]. Still, in contrast to tumor cells, immune cells may not inherently have a strong metabolic flexibility similar to that of tumor cells, which would result in an overall bias toward an immunosuppressive state in the TME, thus promoting the malignant process [5–7].

Tumors are not only aggregates of malignant cells but also well-organized complex ecosystems [8]. Cancer cells communicate directly or indirectly with the surrounding cellular microenvironment and evolve features to promote their survival [9–11]. The dilemma of tumor cells thrives while cytotoxic immune cells are hindered in the TME, which has been a challenge in cancer immunotherapy [12]. However, a recent discovery suggests that the metabolic dominance of tumor cells could be reprogrammed by cytotoxic immune cells, thereby reversing inequalities in the TME which benefits immune cells. Poznanski et al. showed that natural killer (NK) cells with Warburg metabolism and substrate flexibility not only maintained metabolic adaptability but also significantly enhanced tumor-killing capacity under unfavorable conditions of TME [13].

Following extensive research in cancer metabolism during the previous two decades, the latest progress in immunometabolism has further revealed promising signs of metabolic targets modulating anti-cancer immunity. The role of immune signaling networks in immunometabolism is a promising area of research with extensive impact on the therapy of cancer [3]. We focus on the key targets of each of these critical signaling pathways, the upstream and downstream signaling molecules and their mediated cell death as well as specific immune responses, and the clinical studies of small molecule inhibitors or activators of key targets. In addition, some immunometabolic checkpoints that have been reported to play important regulatory roles are also included. Therefore, this review provides essential information about molecular signaling in the field of immunometabolism and targeting strategies in oncology research, as well as highlights how this field drives advances in the therapy of human tumors.

## Metabolic reprogramming in tumor cells

Studies have shown that metabolic reprogramming of tumor cells confers the potential for cancer cells to grow and proliferate in nutrient-deficient TME [14]. One such change in the metabolic pattern of tumor cells was first discovered in 1930 by the German biochemist Otto Warburg, which implied that most tumor cells do not generate energy by the conventional efficient tricarboxylic acid cycle, but instead supply themselves with energy through glycolysis, which is relatively inefficient in terms of energy production [15, 16]. Fast-growing malignant cells usually have a 200-fold higher glycolytic rate than the normal tissue, even in well-oxygenated environments [17]. Such reprogrammed cellular metabolism is now considered a hallmark of cancer [18, 19].

The heterogeneity of tumors and the high demand for nutrients give them a complex metabolic pattern. Besides relying heavily on glycolysis for energy, neoplastic cells promote self-proliferation using glutamine, serine, arginine, fatty acids, and lipids to maintain the harsh anabolic demands and energy productivity [20, 21].

## Metabolic programs in immune cells

Adapted to different tissue environments, immune cells in TME develop specific metabolic characteristics [22, 23]. Different types of immune cells have specific nutritional requirements, using T-cell subsets as well-characterized examples for metabolic adaptation in TME [24]. Helper T cells and effector T cells conform to the “Warburg effect” by taking up large amounts of glucose and accelerating glycolysis while also increasing the rate of oxidative phosphorylation and consuming more glutamine [25–27]. In contrast to these two types of cells, regulatory T cells and memory T cells continue to derive most of the energy from the oxidative phosphorylation process, even after activation, when they preferentially consume fatty acids rather than amino acids and glucose [28, 29].

Metabolic dysfunction of T cells may result in a certain loss of immune function [30]. For instance, Inhibition of pyruvate dehydrogenase kinase, a positive regulator of aerobic glycolysis, disrupts the balance between Teffs and Tregs in the CD4^+^ T-cell subset, decreasing the inflammatory capacity of Th17 cells and promoting Treg cell production [31]. It has been suggested that regulation or reprogramming of metabolic alterations in tumor-infiltrating T cells may represent a potential strategy to revitalize dysfunctional T cells for cancer therapy [32]. In summary, the integration of the metabolic activity of T cells with the functional requirements of each T-cell lineage is an essential aspect of maintaining immune homeostasis and function.

## Crosstalk between immune and metabolic signaling in TME

The constant crosstalk between immune and metabolic signals in TME allows immune and tumor cells with different metabolic patterns. The metabolism of tumor cells causes a large deficiency of nutritional substrates, including glucose and glutamine, in the TME, which results in abnormal metabolism and function of the immune cell population surrounding T cells [7, 33]. Based on the continuous crosstalk between immune and metabolic signals in TME and the effect of metabolic pathways in immune cells, integrating immunometabolic signaling pathways with phosphatidylinositol-3 kinase (PI3K)-protein kinase B (AKT/PKB), mechanistic target of rapamycin (mTOR) and liver kinase B1–5′ AMP-activated protein kinase (LKB1-AMPK) as the central link between immune signaling and metabolic pathways considerably influences tumor progression.

PI3K/Akt/mTOR signaling is one of the most critical intracellular signaling pathways controlling essential cellular functions, but key components of the pathway are frequently dysregulated in a variety of cancers [34]. For example, PI3K signaling is overactivated in breast cancer, inhibition of PI3K reduces the incidence of triple-negative and estrogen receptor-positive breast cancer, and in advanced and metastatic breast cancer, PI3KCA mutations may lead to chemoresistance and poor prognosis [35, 36]. AMPK, as the central metabolism that controls glucose and lipid metabolism, constitutes the most important signaling pathway with LKB1 in response to nutrient and intracellular energy changes. A study suggests that LKB1-AMPK axis inhibition determines esophageal squamous cell carcinoma cell fate from cellular senescence to glutamine-addicted survival [37]. With the deepening of immunometabolic studies, it has been shown that PI3K/Akt/mTOR and LKB1-AMPK, as core immunometabolic signaling pathways, can largely influence tumor progression in the continuous crosstalk between immune and metabolic signals [38].

## PI3K-Akt signaling pathway

**Fig. 1:**
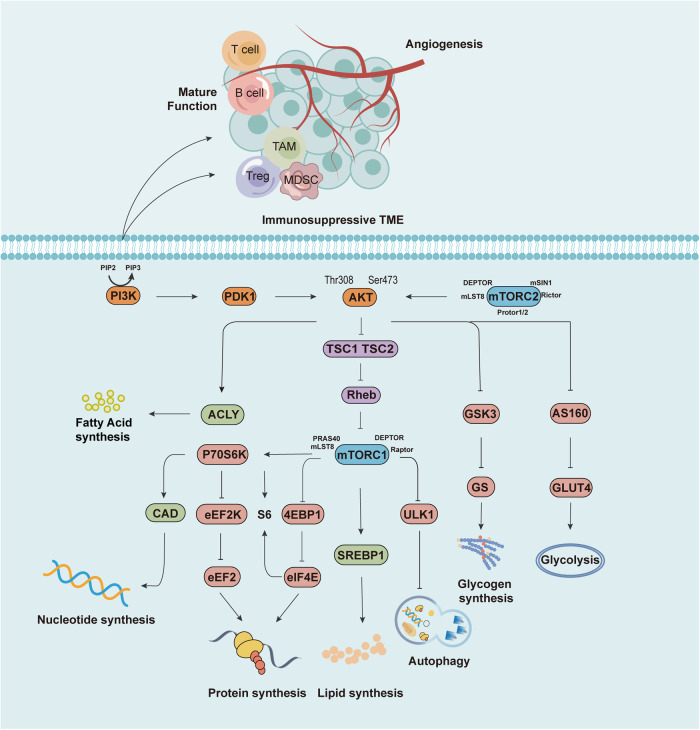
PI3K/AKT/mTOR signaling in immunometabolism. Upon receipt of upstream signals, the PI3K/AKT/mTOR pathway is activated by phosphorylation, acting on a series of downstream signaling molecules to promote the anabolism of fatty acids, nucleotides, proteins, lipids, glycogen, and effector functions of immune cells.

The PI3K/AKT/mTOR pathway tends to be overactivated by cancer, which is considered a promising therapeutic target [39]. PI3K is rapidly activated upon receiving an upstream signal stimulus, affecting a series of downstream targets, including AKT, mTOR, glycogen synthase kinase-3 (GSK3), ATP-citrate lyase (ACLY), etc., performing roles by increasing anabolism and decreasing catabolism (Fig. 1). As the most important signaling pathway in cellular immunometabolism, understanding PI3K signaling-led metabolic reprogramming provides insight into cancer therapeutic potential of pathway inhibitors [40].

## PI3K

PI3K is an intracellular phosphatidylinositol kinase in a dimeric structure with serine/threonine (Ser/Thr) kinase active [41]. According to the structure and substrate specifics, PI3K is classified into classes I, II, and III [42]. The type IA PI3K catalytic subunit includes three proteins, p110α, p110β, and p110δ, and the type IB PI3K catalytic subunit, p110γ [43, 44]. The signaling pathway consisting of PI3K and its downstream signaling molecules are essential for cell viability, cycling, metabolism, and other physiological functions in mammals [45]. This also allows signaling pathways mediated by PI3K relevant to multiple disease areas such as tumor therapy, cellular metabolism, inflammation genesis, and immunity [46]. PI3K is activated by signals from receptor tyrosine kinases or G protein-coupled receptors [47]. Upon receipt of an upstream signal, PI3K phosphorylates phosphatidylinositol-4,5-bisphosphate (PIP2), producing large amounts of phosphatidylinositol-3,4,5-trisphosphate (PIP3), thereby recruiting protein kinase-1 (PDK1), phosphorylating the 308 site that activates AKT [48, 49]. In addition, PDK1 can also activate mTORC2, which activates AKT signaling downstream by acting on the tryptophan 473 sites.

As PI3K is widely associated with many intra-organic processes, they have a close influence on both cellular metabolic processes and the immune system, and the activation of PI3K can be involved in multiple biological processes of immune cell development, activation or migration [50]. On one hand, PI3K activity affects the maturation and function of T and B cells [51]. Klaus Okkenhaug and colleagues found loss of PI3Kδ function in B and T cells in a mouse model of p110δ mutation, which leads to impaired T and B-cell antigen receptor signaling and diminished immune responses in vivo, and these mutations in p110δ contribute to the combined immunodeficiency syndrome [52]. PI3K activity also affects neutrophils, macrophages, NK cells, and dendritic cells (DCs) [53, 54]. First, the generation of reactive oxygen species (ROS) by neutrophils to kill microorganisms is dependent on p110γ and p110βPI3K [55]. Second, NK cell maturation and function are critically related to p110δ and p110γ, including cytokine secretion and cytotoxicity. Third, PI3K creates a TME favorable for tumor growth mainly by promoting tumor-associated macrophages (TAMs), regulatory T cells (Tregs), and myeloid-derived suppressor cells (MDSCs), where PI3Kδ dominates the immunosuppressive function of Tregs and MDSCs [56]. In addition, PI3K signaling is specifically vital in tumor angiogenesis. Given the multiple effects of PI3K on TME, PI3K inhibitors promote the proliferation of anti-tumor cells and the infiltration of immune cells to a certain extent, facilitating a positive immunomodulatory efficacy.

PI3K is invariably widely overactivated in cancer and immune dysregulation, manifested by a significant correlation between enhanced tumor microvessel density and increased invasiveness of tumor cells. This has led researchers to look at developing therapeutic PI3K inhibitors, and despite drug resistance and tolerance issues, some PI3K inhibitors have been approved for marketing. Initial studies always focused on pan-PI3K inhibitors. However, as research progressed, excessive toxicities were an important factor limiting the development of such inhibitors, with Bayer’s targeting PI3Kα and PI3Kδ Copanlisib coming last and being approved for recurrent follicular lymphoma in 2017 [57, 58]. And of course, Duvelisib is a PI3Kδ and PI3Kγ inhibitor for lymphoma, which was launched in 2018 [59]. PI3K inhibitors with subtype specificity stand out in this research dilemma, mainly alpelisib, a PI3Kα inhibitor launched in 2019 [60–62]. Idelalisib, a PI3Kδ inhibitor for chronic lymphatic leukemia, launched in 2014 [63, 64], and many drugs in clinical studies, PI3K-mTOR, and other multi-target inhibitors are listed below. Specifically, PI3K inhibitors exert efficacy in the following ways. For example, first, since a lack of PI3Kδ and PI3Kγ is associated with impaired immune responses and B-cell development, inhibition of signaling from B-cell receptors can be useful in B-cell lymphoma [65]. Second, some studies have used PI3Kγ inhibitors IPI-549 and silymarin to target tumor-associated fibroblasts to exert anti-cancer activities, as evidenced by significant reductions in Treg and MDSCs, as well as suppression of angiogenesis and the formation of collagen in tumor tissue [66]. Third, given that PI3Kδ dominates the immunosuppressive function of Tregs and MDSCs as described above, PI3Kδ inhibitors may contribute to a positive immune environment and promote cytotoxic T-cell responses [56, 67]. Overall, the reliance of regulatory immune cells upon the PI3K pathway could be treated with PI3K inhibitors to release immunosuppression and restore CD8^+^ T-cell activity (Table 1).

**Table 1:** Potential inhibitors of the PI3K/AKT/mTOR pathway available for targeting PI3K.

Target	Drug	Phase/status	Tumor type	Treatment	ClinicalTrials. govIdentifier
PI3K/AKT/mTOR inhibitors					
PI3Kα	Alpelisib	FDA approved for marketing	Breast cancer	With Fulvestrant	
	Taselisib	II Ongoing	Advanced lymphoma Advanced malignant Solid neoplasm Hematopoietic and lymphoid cell neoplasm …	Monotherapy	NCT04439175
		II Completed	Recurrent squamous cell lung carcinoma Stage IV squamous cell lung carcinoma	Monotherapy	NCT02785913
		I Ongoing	Metastaticbreast cancer Recurrent breast cancer	With Trastuzumab emtansine, Paclitaxel Pertuzumab, Trastuzumab	NCT02390427
		II Completed	Breast cancer	With Letrozole,	NCT02273973
		II Ongoing	Advanced malignant Solid neoplasm Bladder carcinoma Breast carcinoma Cervical carcinoma…	With Adavosertib, Afatinib, Afatinib Dimaleate…	NCT02465060
	Inavolisib	II Ongoing	HER2-positive breast cancer	With PHESGO	NCT05306041
		III Ongoing	Breast cancer	With Placebo, Palbociclib, Fulvestrant	NCT04191499
		I Ongoing	Metastatic colorectal cancer	With Bevacizumab, Cetuximab, SY-5609, Atezolizumab, Tiragolumab	NCT04929223
		II Ongoing	Breast cancer Triple-negative breast cancer	With Capecitabine, Talazoparib, Atezolizumab	NCT04849364
		II Ongoing	Early-stage breast cancer	With Atezolizumab, Ipatasertib, Olaparib	NCT05332561
		I/II Ongoing	Inoperable, locally advanced or metastatic, ER-positive breast cancer	With Giredestrant, Abemaciclib, Ipatasertib…	NCT04802759
	Serabelisib (TAK-117/MLN1117)	I Ongoing	Advanced solid tumor PIK3CA mutation PTEN loss of function mutation	With Nab-paclitaxel	NCT05300048
		II Completed	Endometrial neoplasms	With Paclitaxel, Sapanisertib	NCT02725268
	MEN1611	I/II Ongoing	Metastatic colorectal cancer	With Cetuximab	NCT04495621
		I Ongoing	Advanced or metastatic breast cancer	With Trastuzumab, Fulvestrant	NCT03767335
	CYH33	I Ongoing	Ovarian cancer Breast cancer Solid tumor Prostate cancer Endometrial cancer	Monotherapy	NCT04586335
		II Ongoing	Ovarian cancer Recurrent cancer	Monotherapy	NCT05043922
		I Ongoing	Advanced breast cancer	With Fulvestrant, Letrozole, Palbociclib	NCT04856371
PI3Kβ	GSK2636771	II Ongoing	Advanced lymphoma Advanced malignant Solid neoplasm Hematopoietic and lymphoid cell neoplasm Refractory lymphoma Refractory malignant	Monotherapy	NCT04439188
		II Ongoing	Advanced lymphoma Advanced malignant Solid neoplasm Hematopoietic and lymphoid cell neoplasm Refractory lymphoma Refractory malignant	Monotherapy	NCT04439149
		I/II Ongoing	Melanoma and other malignant neoplasms of skin Metastatic melanoma	With Pembrolizumab	NCT03131908
	SAR260301	I/Ib Completed	Neoplasm malignant	With Vemurafenib	NCT01673737
	AZD8186	I Ongoing	Advanced breast carcinoma Advanced malignant Solid neoplasm Advanced prostate carcinoma …	With Docetaxel	NCT03218826
		I/II Ongoing	Solid tumor Stomach cancer	With Paclitaxel	NCT04001569
		I/II Ongoing	Solid tumor Stomach cancer	With Alpelisib, Paclitaxel	NCT04526470
PI3Kγ	IPI549 (Eganelisib)	II Ongoing	Bladder cancer Urothelial carcinoma Solid tumor Advanced cancer	With Nivolumab, Placebos	NCT03980041
		II Ongoing	Head and neck squamous cell carcinoma HPV-related carcinoma HPV-related malignancy HPV-related squamous cell carcinoma	Monotherapy	NCT03795610
PI3Kδ	Idelalisib	FDA approved for marketing	Chronic lymphocytic leukemia Follicular lymphoma Small lymphocytic leukemia	With Rituxan, Monotherapy	
	Umbralisib	I/II Ongoing	Mantle cell lymphoma	With Ublituximab	NCT04692155
		II Ongoing	Lymphoma Follicular lymphoma Marginal zone lymphoma Marginal zone B-cell lymphoma	With Rituximab	NCT03919175
		II Completed	Waldenstrom macroglobulinemia	Monotherapy	NCT03364231
		II Ongoing	Chronic lymphocytic leukemia	With Ublituximab	NCT04149821
		I Completed	Chronic lymphocytic leukemia Richter syndrome	With Ublituximab, TG-1501	NCT02535286
		II Ongoing	Chronic lymphocytic leukemia	With Ublituximab, Ibrutinib, Venetoclax, Acalabrutinib Oral Capsule	NCT04016805
		II/III Ongoing	Chronic lymphocytic leukemia Small lymphocytic lymphoma	With Ublituximab, Venetoclax	NCT03801525
		II Ongoing	Chronic lymphocytic leukemia Small lymphocytic lymphoma Relapsed chronic lymphocytic leukemia Refractory chronic lymphocytic leukemia	With Acalabrutinib, Ublituximab	NCT04624633
		I Ongoing	B-cell non-Hodgkin lymphoma Relapsed B-cell non-Hodgkin lymphoma Refractory B-cell non-Hodgkin lymphoma	With Loncastuximab Tesirine, Gemcitabine, Lenalidomide, Polatuzumab Vedotin	NCT04970901
		I Ongoing	Non-Hodgkin lymphoma Chronic lymphocytic leukemia	With TG1701, Ublituximab	NCT03671590
		I Ongoing	Chronic lymphocytic leukemia B-cell non-Hodgkin lymphoma	With TGR1202, Pembrolizumab	NCT03283137
		I/II Ongoing	Chronic lymphocytic leukemia Waldenstrom macroglobulinemia Mantle cell lymphoma Marginal zone lymphoma B-cell lymphoma	With Pirtobrutinib, Venetoclax, Rituximab	NCT03740529
	Parsaclisib (INCB50465)	II Ongoing	Lymphoma	Monotherapy	NCT04434937
		I Completed	Advanced malignancies	Monotherapy	NCT04831944
		II Ongoing	Lymphoma	Monotherapy	NCT03126019
		II Completed	Lymphoma	Monotherapy	NCT02998476
		I Ongoing	Lymphoma	Monotherapy	NCT03314922
		III Ongoing	Myelofibrosis Primary myelofibrosis Post essential thrombocythemia myelofibrosis Post polycythemia vera myelofibrosis	With Ruxolitinib, Placebo	NCT04551053
		I/II Ongoing	Chronic lymphocytic leukemia Non-Hodgkin lymphoma	With Tafasitamab	NCT04809467
		II Ongoing	Lymphoma	Monotherapy	NCT03235544
		I Completed	Advanced malignancies	Monotherapy	NCT04831996
		I/II Ongoing	Peripheral T-cell lymphoma	With Chidamide	NCT05083208
		I Completed	B-cell lymphoma	With Rituximab, Bendamustine, Ibrutinib	NCT03424122
	Zandelisib (ME-401)	II Ongoing	Follicular lymphoma Non-Hodgkin Lymphoma Marginal zone lymphoma	Monotherapy	NCT03768505
		III Ongoing	Follicular lymphoma Non-Hodgkin lymphoma Marginal zone lymphoma	With Rituximab, Bendamustine, CHOP	NCT04745832
		II Ongoing	Chronic lymphocytic leukemia	With Rituximab, Venetoclax	NCT05209308
	Linperlisib (YY-20394)	I Ongoing	Advanced solid tumor	Monotherapy	NCT05429398
		II Ongoing	Peripheral T/NK cell lymphoma	Monotherapy	NCT05274997
		I/II Ongoing	Peripheral T-cell lymphoma	With Azacitidine Injection, Dasatinib, Tucidinostat, SHR2554, Apatinib, Camrelizumab	NCT05559008
	IOA-244	I Ongoing	Solid tumor, adult Non-Hodgkin lymphoma, adult Non-small-cell lung cancer Myelofibrosis Uveal melanoma	With Avelumab Injection, Cisplatin, Pemetrexed, Ruxolitinib	NCT04328844
Pan-PI3K	Copanlisib	FDA approved for marketing	Follicular lymphoma	Monotherapy	
	Buparlisib (BKM120)	III Ongoing	Head and neck cancer	With Paclitaxel	NCT04338399
		I Ongoing	Mantle cell lymphoma Follicular lymphoma Diffuse large B-cell lymphoma	With Ibrutinib	NCT02756247
	Pictilisib (GDC-0941)	II Completed	Non-small cell lung cancer	With Placebo, Bevacizumab, Carboplatin, Paclitaxel	NCT01493843
		I Completed	Breast cancer	With Bevacizumab, Letrozole, Paclitaxel, Trastuzumab	NCT00960960
		II Completed	Breast cancer	With Placebo, Paclitaxel	NCT01740336
	SF1126	I Completed	Advanced or metastatic solid tumors cancer	Monotherapy	NCT00907205
	Pilaralisib (XL147)	II Completed	Endometrial cancer Endometrial neoplasms	Monotherapy	NCT01013324
		I/II Completed	Breast cancer Breast neoplasms	With Trastuzumab, Paclitaxel	NCT01042925
		I Completed	Non-small cell lung cancer	With Erlotinib	NCT00692640
	PX-866	II Completed	Prostate cancer	Monotherapy	NCT01331083
		I Completed	Advanced solid tumors	Monotherapy	NCT00726583
		II Completed	Glioblastoma	Monotherapy	NCT01259869
		I/II Completed	Non-small cell lung cancer, squamous cell carcinoma of the head and neck	With Docetaxel	NCT01204099
	ZSTK474	I Completed	Neoplasms	Monotherapy	NCT01280487
	CH5132799	I Completed	Solid tumors	Monotherapy	NCT01222546
Dual PI3Kδ /PI3Kγ	Duvelisib (Copiktra)	FDA approved for marketing	Chronic lymphocytic leukemia Small lymphocytic lymphoma	Monotherapy	
	Tenalisib (RP6530)	I/II Completed	T-cell lymphoma	With Romidepsin	NCT03770000
		II Completed	Non-Hodgkin lymphoma	Monotherapy	NCT03711578
		II Ongoing	Locally advanced breast cancer Metastatic breast cancer	Monotherapy	NCT05021900
		II Ongoing	Peripheral T-cell lymphoma	Monotherapy	NCT05239910
Dual PI3Kα/PI3Kδ	TQ-B3525	I/II Ongoing	Non-small-cell Lung cancer	With Osimertinib Mesylate tablets	NCT05284994
		II Ongoing	Peripheral T-cell lymphoma	Monotherapy	NCT04615468
		I/II Ongoing	Relapsed/refractory chronic lymphocytic leukemia/small lymphocytic lymphoma	Monotherapy	NCT04808570
		II Ongoing	Diffuse large B-cell lymphoma	Monotherapy	NCT04610970
		II Ongoing	Relapsed/refractory follicular lymphoma	Monotherapy	NCT04324879
		II Ongoing	Advanced endometrial cancer, cervical cancer, and ovarian cancer	Monotherapy	NCT04836663

## AKT

AKT, also called PKB, is a Ser/Thr kinase consisting of three allomorphic forms [68]. When the Thr308 and Ser473 sites of AKT are fully activated by phosphorylation of PIP3 and mTORC2, thereby affects a series of downstream substrates, resulting in increased anabolism and decreased catabolism [69]. Specifically, first, AKT inhibits the negative regulatory effect of AKT substrate of 160 kDa (AS160) on glucose transporter 4 (GLUT4), allowing cells to translocate GLUT4-containing vesicles and permit glucose to enter the cell for glycolysis [70]. Second, AKT inhibits GSK3, which relieves the inhibition of glycogen synthase and promotes glycogen synthesis, allowing cells to take up glucose more easily. Third, AKT phosphorylates and activates ACLY to promote fatty acid synthesis. Fourth, AKT inhibits tuberous sclerosis complex 1/2 (TSC1/2) through phosphorylation and unbinds the Ras homolog enriched in the brain (RHeb), allowing RHeb to activate mTORC1 [71].

AKT affects the immune system in two major ways. First, the Akt pathway regulates the activation phenotype of macrophages and modulates macrophage responses through inflammatory and metabolic signaling [72]. Macrophages are classified into M1-type and M2-type [73]. M1-type macrophages are involved in positive immune responses and perform immune surveillance functions. In contrast, the weak antigen-presenting capacity and the secreted suppressive cell factors of M2-type macrophages mediate immune suppression, in which AKT may function. Second, AKT acts as a protector in regulating the evolution of memory CD8^+^ T-cell responses. As found by Anne Rogel and others, AKT has a crucial role in the immune surveillance of memory CD8^+^ T cells, as demonstrated that the deficiency of AKT affects the survival of effector CD8^+^ T cells on conversion to memory CD8^+^ T cells, leading to a reduction in the number of memory CD8^+^ T cells and weakening secondary immunity. Also, weakened AKT leads to a deficiency of certain tumor-fighting effector cell types in memory CD8^+^ T cells, which leads to a reduced ability and lessened effectiveness against tumors [74].

AKT directly influences numerous tumorigenic processes. Given the great importance of AKT, it is a promising therapeutic target, and several AKT inhibitors are under clinical research [75]. Currently, there are allosteric AKT inhibitors in clinical trials, such as MK-2206, BAY1125976, and miransertib, as well as ATP-competitive inhibitors, such as capivasertib and ipatasertib [76, 77]. ATP-competitive inhibitors directly target the kinase structural domain to inhibit its activity. Activating mutations and abnormal expression of the AKT pathway are related to the genesis of many types of cancers, such as breast and lung cancers [78, 79]. A natural product modifier from Brassica vegetables, 3-chloroacetylindole, established as a valid non-competitive AKT1 and AKT2 inhibitor, proved to suppress colorectal cancer cell growth and trigger apoptosis both in vivo and in vitro [80] (Table 2).

**Table 2:** Potential inhibitors of the PI3K/AKT/mTOR pathway available for targeting AKT.

Target	Drug	Phase/Status	Tumor type	Treatment	ClinicalTrials. govIdentifier
PI3K/AKT/mTOR Inhibitors					
** AKT**	MK-2206	II Completed	Colorectal neoplasms	With AZD624	NCT01333475
		II Completed	PANCREAS Neuroendocrine	Monotherapy	NCT01169649
		II Completed	Ovarian sarcoma Recurrent fallopian tube carcinoma, recurrent ovarian carcinoma Recurrent primary peritoneal carcinoma	Monotherapy	NCT01283035
		II Completed	Recurrent nasopharyngeal carcinoma	Monotherapy	NCT01370070
		II Completed	Diffuse large B-cell lymphoma	Monotherapy	NCT01481129
		I/II Completed	Chronic lymphocytic leukemia Recurrent small Lymphocytic lymphoma Refractory chronic Lymphocytic leukemia	With Bendamustine Hydrochloride, Rituximab	NCT01369849
		II Completed	Pancreatic acinar cell carcinoma Pancreatic ductal Adenocarcinoma Recurrent pancreatic carcinoma	With Fluorouracil, Oxaliplatin, Selumetinib	NCT01658943
		II Ongoing	Carcinoma, non-small-cell lung Carcinoma, small-cell lung carcinoma, thymic	With AZD6244, Lapatinib, Erlotinib, Sunitinib	NCT01306045
		II Completed	Lung cancer	With Erlotinib, AZD6244, Sorafenib	NCT01248247
		II Ongoing	Breast neoplasms Breast cancer Angiosarcoma TNBC-triple-negative breast cancer…	With AMG 386, AMG 479, MK-2206, AMG 386 and Trastuzumab, T-DM1 and Pertuzumab…	NCT01042379
	BAY1125976	I Completed	Neoplasms	Monotherapy	NCT01915576
	TAS‑117	II Completed	Solid tumor, adult	Monotherapy	NCT03017521
		II Ongoing	Advanced or metastatic solid tumors irrespective of gene alterations Advanced or metastatic solid tumors with germline PTEN inactivating mutations	Monotherapy	NCT04770246
	MSC2363318A	I Completed	Solid tumor	With Trastuzumab, Tamoxifen	NCT01971515
	AZD5363 (Capivasertib)	I Ongoing	Solid tumor, adult	With Olaparib, Durvalumab	NCT03772561
		I Ongoing	Breast cancer Prostate cancer Advanced solid tumors	With Enzalutamide, Fulvestrant	NCT03310541
		I/II Completed	Prostate cancer	With Placebo	NCT02121639
		II Completed	Advanced gastric cancer	With Paclitaxel	NCT02451956
		I/II Ongoing	Estrogen receptor-positive breast cancer	With Fulvestrant	NCT01992952
		I/II Completed	Advanced or metastatic breast cancer ER+ve advanced or metastatic breast cancer	With Paclitaxel	NCT01625286
		I Completed	Prostate cancer	With Capivasertib, Enzalutamide, Abiraterone	NCT04087174
		I Completed	Advanced solid tumors	With Capivasertib, Paclitaxel	NCT04742036
		III Ongoing	Triple-negative breast neoplasms	With Capivasertib, Paclitaxel	NCT03997123
		II Ongoing	Intracranial meningioma Recurrent meningioma NF2 gene mutation	With Vismodegib, GSK2256098, Capivasertib, Abemaciclib	NCT02523014
		II Ongoing	Advanced breast cancer	With Fulvestrant, Neratinib, AZD5363, Olaparib, AZD6738	NCT03182634
		I/II Ongoing	Triple-negative breast neoplasms	With Durvalumab, Capivasertib, Oleclumab, Paclitaxe, Trastuzumab deruxtecan, Datopotamab deruxtecan	NCT03742102
		II Ongoing	Anatomic stage IV breast cancer AJCC v8 Metastatic triple-negative breast carcinoma	With Capivasertib, Ceralasertib, Olapari, Selumetinib	NCT03801369
	Uprosertib	I/II Ongoing	Hematopoietic and lymphoid cell neoplasm Locally advanced malignant solid neoplasm Locally advanced melanoma Metastatic malignant solid neoplasm…	With Dabrafenib Mesylate, Trametinib Dimethyl Sulfoxide	NCT01902173
	Ipatasertib	I Ongoing	Metastatic breast cancer	With Trastuzumab, Pertuzumab	NCT04253561
		I Ongoing	Head and neck carcinoma of unknown primary Locally advanced head and neck squamous cell carcinoma…	With Cisplatin	NCT05172245
		I Ongoing	Solid tumors	Monotherapy	NCT04341259
		II Ongoing	NSCLC stage IV NSCLC stage IIIB	Monotherapy	NCT04467801
		II Ongoing	Triple-negative breast cancer	With Capecitabine, Eribulin, Carboplatin, Gemcitabine	NCT04464174
		I/II Ongoing	Endometrial endometrioid adenocarcinoma	With Megestrol Acetate	NCT05538897
		II Ongoing	Breast cancer	With Fulvestrant, Palbociclib, CDK4/6 Inhibitor	NCT04920708
		I Ongoing	Castration-resistant prostatic cancer	With Atezolizumab, Docetaxel	NCT04404140
		II Ongoing	Head and neck squamous cell carcinoma	With Pembrolizumab	NCT05172258
		I/II Ongoing	Solid tumor Glioblastoma multiforme Prostate cancer Metastatic	With Atezolizumab	NCT0367378
		II Completed	Breast cancer	With Paclitaxel	NCT02301988
		II Ongoing	Locally advanced malignant Solid neoplasm Metastatic malignant solid Neoplasm Unresectable malignant solid Neoplasm	With Paclitaxel	NCT05554380
		III Ongoing	Breast cancer	With Paclitaxel, Placebo	NCT03337724
		II Completed	Gastric cancer	With Leucovorin, 5-Fluorouracil, Oxaliplatin	NCT01896531
		III Ongoing	Breast cancer	With Fulvestrant, Palbociclib	NCT04060862
		III Ongoing	Breast cancer	With Fulvestrant	NCT04650581
		III Ongoing	Triple-negative breast cancer	With Atezolizumab, Paclitaxel	NCT04177108
		II Ongoing	Gastric adenocarcinoma	With Atezolizumab, Bevacizumab	NCT04739202
		II Ongoing	Breast cancer	With Atezolizumab Injection, Pertuzuma, Trastuzumab, Bevacizumab,	NCT05180006
		II Ongoing	Breast cancer Estrogen receptor-positive breast cancer	With Atezolizumab, Cobimetinib, Bevacizumab	NCT03395899
		II Ongoing	Triple-negative breast cancer Residual cancer Circulating tumor DNA	With Atezolizumab, Sacituzumab, govitecan	NCT04434040
		I/II Ongoing	Endometrial cancer	With Atezolizumab, Bevacizumab, Talazoparib, Trastuzumab, emtansine, Tiragolumab	NCT04486352
		I/II Ongoing	Carcinoma, non-small-cell lung	With Atezolizumab, Cobimetinib, RO6958688, Docetaxel, CPI-444, Pemetrexed, Carboplatin, Gemcitabine…	NCT03337698

## mTOR

Mammalian targets proteins of rapamycin, mTOR, including mTORC1 and mTORC2, which is the major regulators of cellular metabolism. Multiple studies have shown that mTORC1 activation is associated with metabolic reprogramming [81]. First, mTORC1 phosphorylation activates p70 ribosomal protein S6 kinase (P70S6K), the most important signaling hub downstream of it signaling pathway, to promote intracellular pyrimidine synthesis, peptide translation synthesis, peptide chain extension, and other pathways leading to increased protein synthesis. Second, mTORC1 could also synergistically increase protein synthesis by inhibiting 4E-binding protein 1 (4EBP1) through phosphorylation so that eukaryotic translation initiation factor 4E (eIF4E) could activate the S6 ribosomal subunit and activate ribosomes [82, 83]. Finally, mTORC1 inhibits cellular autophagy by inhibiting unc-51-like kinase-1 (ULK1), an important initiator that controls autophagosome production and maturation, through phosphorylation.

mTOR has a variety of immunological functions, primarily modulating the differentiation and function of immune cells and also having crucial functions in memory cell development. On one hand, mTOR regulates the differentiation, survival, and metabolic reprogramming of T-cell subsets [84]. On the other hand, mTOR determines the proliferation and maturation of Treg, Th17 cells, and NK cells as well as influences effector function and cytotoxicity [85]. Additionally, mTOR serves a crucial role in regulating cell death, mainly in autophagy, ferroptosis, and scorching death. Given the dual role of autophagy in suppressing cancer at an early stage while maintaining tumor metabolism, growth, and survival promoting tumorigenesis at a later stage. Therefore, inhibiting autophagy with mTOR inhibitors at specific times may increase the metabolic stress on cancer cells to facilitate cell death. The oncogenic activation of the PI3K/AKT/mTORC1 pathway can also inhibit ferroptosis in cancer cells through downstream SREBP1/scd1-mediated adipogenesis, so the combination of mTOR inhibitors and other ferroptosis inducers for cancer treatment may be an excellent therapeutic target [86]. Experiments by Wang, Y. demonstrated a novel mechanism by which mTORC2 signaling promoted the long-lasting maintenance of memory CD4^+^ T cells by inhibiting the onset of ferroptosis [87]. This study further demonstrated the major form of memory CD4^+^ T-cell ferroptosis in the presence of mTORC2 deficiency through knockdown and overexpression experiments of GPX4, a key enzyme of the ferroptosis pathway. A new study conducted by Evavold and others found that mTORC1 promoted gasdermin D-mediated inflammatory cell death by controlling ROS production in mitochondria [88, 89].

Dysregulated mTOR activity can be found in a variety of cancers, including prostate, breast, lung, melanoma, bladder, brain, and kidney cancers, leading to mTOR as a critical therapeutic target. The mTOR inhibitor blocks signaling producing positive effects of anti-inflammatory, anti-tumor cell proliferation, and inducing apoptosis. Currently, mTOR inhibitors proceed to the third generation. Sirolimus (rapamycin), everolimus, tesilimus, lidaformycin, and zotamox are the first-generation inhibitors of mTOR, and they are called rapamycin and its derivatives. First-generation mTOR inhibitors, mainly inhibit the complex mTORC1, which may lead to compromised negative feedback on the PI3K signaling pathway, which in turn enhances the phosphorylation activity of AKT, making patients susceptible to drug resistance with the drug. Second-generation inhibitors competitively inhibit mTOR kinases, including mTORC1 and mTORC2. This class of inhibitors blocks the feedback regulation of AKT activation formation caused by mTORC1 inhibitors, so this class of drugs has a stronger inhibitory effect than mTORC1 inhibitors. Rapalink-1, a third-generation mTOR inhibitor, is a linkage of first-and second-generation mTOR inhibitors that can target both targets on the mTOR enzyme. The functions of mTOR signaling in immune cell regulation are diverse, on the one hand promoting T-cell accumulation and clearance in tumors, yet on the other hand, mediating tumor malignancy development and immune evasion. Tumor cells utilize this pathway for vicious progression potentially providing opportunities for the development of T-cell-based immunotherapies. Similarly, mTOR inhibitors can greatly promote or inhibit T-cell chemokine-mediated chemotaxis in TME or inflammation. Although the anti-tumor effect of mTOR signaling and chemokine/receptor axis in mediating immune cells or tumor cells is two-sided, whose effect it has remains an open question (Tables 3 and 4).

**Table 3:** Potential inhibitors of the PI3K/AKT/mTOR pathway available for targeting mTOR.

Target	Drug	Phase/Status	Tumor type	Treatment	ClinicalTrials. govIdentifier
PI3K/AKT/mTOR Inhibitors					
mTOR	Everolimus	FDA approved for marketing	Advanced-stage renal cancer, advanced-stage HR+breast cancer in postmenopausal women in combination with exemestane, well-differentiated neuroendocrine tumors, renal angiomyolipoma, tuberous sclerosis complex, Subependymal giant cell astrocytoma, tuberous sclerosis complex	Monotherapy With exemestane	
	Temsirolimus	FDA approved for marketing	Advanced renal cell carcinoma,	Monotherapy	
	Rapamycin	I Completed	Bladder cancer	Monotherapy	NCT02753309
		II Ongoing	Non-muscle invasive bladder cancer	With Encapsulated Rapamycin	NCT04375813
		I/II Ongoing	Advanced soft tissue sarcoma, locally advanced soft tissue sarcoma, metastatic soft tissue sarcoma	With Nanoparticle Albumin-Bound Rapamycin, Pazopanib hydrochloride	NCT03660930
		I Ongoing	Advanced malignant solid neoplasm, recurrent brain neoplasm, recurrent malignant solid neoplasm, refractory brain neoplasm	With Dasatinib, Cyclophosphamide	NCT02389309
		IV Ongoing	Uterine fibroids	Monotherapy	NCT03500367
	Ridaforolimus (MK-8669)	I Completed	Cancer, advanced	Monotherapy	NCT01380184
		II Completed	Sarcoma	Monotherapy	NCT01010672
		II Completed	Breast neoplasms	With Dalotuzumab, Exemestane	NCT01605396
	Vistusertib (AZD2014)	II Completed	Diffuse large B-cell lymphoma	With Rituximab	NCT02752204
	AZD8055	I Completed	Glioblastoma multiforme, anaplastic astrocytoma, anaplastic oligodendroglioma, malignant glioma, brainstem glioma	Monotherapy	NCT01316809
	Sapanisertib (MLN0128)	I Completed	Advanced solid tumors	Monotherapy	NCT02197572
		II Ongoing	Non-small cell lung cancer	Monotherapy	NCT05275673
		II Completed	Breast neoplasms	With Fulvestrant	NCT02756364
		I Completed	Malignant solid neoplasm	With Carboplatin, Paclitaxel	NCT03430882
		II Completed	Endometrial neoplasms	With Paclitaxel, MLN1117	NCT02725268
	CC-223 (ATG-008)	I Ongoing	Lymphoma, large B-cell, diffuse	With CC-122, Rituximab, CC-292	NCT02031419
		II Ongoing	Hepatocellular carcinoma	Monotherapy	NCT03591965
	OSI‑027	I Completed	Any solid tumor or lymphoma	Monotherapy	NCT00698243

**Table 4:** Potential inhibitors of the PI3K/AKT/mTOR pathway available for targeting PI3K and mTOR.

Target	Drug	Phase/Status	Tumor type	Treatment	ClinicalTrials. govIdentifier
PI3K/AKT/mTOR Inhibitors					
Dual pan-PI3K and mTOR	Apitolisib	I/II Completed	Prostate cancer	With Abiraterone, Ipatasertib, Prednisone, Prednisolone	NCT01485861
	Dactolisib (BEZ235)	I Completed	Malignant solid tumor	Monotherapy	NCT01343498
	Gedatolisib (PF-05212384, PKI-587)	II Ongoing	HER2-positive breast cancer, metastatic breast cancer	With Herzuma	NCT03698383
		I/II Ongoing	Triple-negative breast cancer	With Talazoparib	NCT03911973
		I Ongoing	Lung cancer squamous cell, solid tumors, head & neck cancer, pancreatic cancer	With Palbociclib	NCT03065062
		III Ongoing	Breast cancer	With Palbociclib, Fulvestrant, Alpelisib	NCT05501886
		I Completed	Triple-negative breast cancer, metastatic breast cancer	With PTK7-ADC	NCT03243331
		I Completed	Breast cancer	With Palbociclib, Letrozole, Fulvestrant	NCT02684032
	Sapanisertib (INK-128, MLN0128, TAK-228)	I Completed	Advanced solid tumors	Monotherapy	NCT02197572
		II Ongoing	Non-small cell lung cancer, squamous non-small-cell lung cancer Squamous non-small cell neoplasm of lung NFE2L2 gene mutation	Monotherapy	NCT05275673
		II Completed	Breast neoplasms	With Fulvestrant	NCT02756364
		I Completed	Recurrent malignant solid neoplasm, refractory malignant solid neoplasm	With Carboplatin, Paclitaxel	NCT03430882
		II Completed	Lung squamous cell carcinoma	Monotherapy	NCT02417701
	BGT226	I/II Completed	Solid tumors, breast cancer, Cowden syndrome	Monotherapy	NCT00600275
	PQR309	I Completed	Lymphoma Non-Hodgkin lymphoma	Monotherapy	NCT03127020
		II Completed	Primary central nervous system lymphoma	Monotherapy	NCT02669511
		I/II Completed	Metastatic breast cancer	With Eribulin	NCT02723877
	LY3023414	I Completed	Neoplasm	Monotherapy	NCT02536586
		II Completed	Endometrial cancer Recurrent endometrial cancer	Monotherapy	NCT02549989
		II Completed	Prostate cancer metastatic	With Enzalutamide	NCT02407054
		I Completed	Advanced cancer Metastatic cancer Non-Hodgkin’s lymphoma, metastatic breast cancer Malignant mesothelioma Non-small cell lung cancer	With Midazolam, Fulvestrant, Pemetrexed, Cisplatin, Letrozole, Abemaciclib	NCT01655225
	PF-04691502	I Completed	Cancer	Monotherapy	NCT00927823
	GDC-0980	II Completed	Renal cell carcinoma	With Everolimus	NCT01442090
		II Completed	Endometrial carcinoma	Monotherapy	NCT01455493
		I Completed	Breast cancer	With Bevacizumab, Paclitaxel	NCT01254526
	Voxtalisib (XL765, SAR245409)	I Completed	Cancer	Monotherapy	NCT00485719
		I Completed	Gliomas	With Temozolomide	NCT00704080
		I Completed	Non-small cell lung cancer	With Erlotinib	NCT00777699
		I Completed	Neoplasm malignant	Monotherapy	NCT01596270
		II Completed	Lymphoma	Monotherapy	NCT01403636
	GSK2126458	I Completed	Solid tumors	Monotherapy	NCT00972686

## GSK3

Glycogen synthase kinase-3 (GSK-3) is also a downstream target of AKT [90, 91]. AKT phosphorylates and inhibits GSK-3, then targets proteasomal degradation to promote glycogen synthesis [92, 93]. Studies now show that GSK-3 is anomalously regimented in various cancers and has oncogenic effects. Thus GSK-3 is emerging as a possible curative candidate for cancer [94, 95]. Although some GSK-3 inhibitors have shown poor efficacy in studies, there is also evidence that they can inhibit the growth of certain cancers [96]. For example, Frank Cichocki and others demonstrated that in the existence of GSK3 inhibitor CHIR99021, the production of tumor necrosis factor-α (TNF-α) and interferon-γ (IFN-γ) by NK cells was significantly increased, which enhanced NK cytotoxicity, fueled immunotherapy of cancer [97]. Furthermore, research has also shown that downregulation of GSK-3 expression using siRNA or inhibition of GSK-3 expression with small molecule inhibitors both downregulate PD-1 levels to augment the killing capacity in CD8^+^ T cells [98].

## ACLY

ATP-citrate lyase (ACLY) is a key enzyme that catalyzes the synthesis of fatty acids and is the major enzyme responsible for the production of acetyl coenzyme A at the cell membrane in many tissues [99, 100]. As is known, malignantly accreting tumor cells exhibit a great demand for lipids and a significant upregulation of fatty acid biosynthetic pathways in various tumor cells [101, 102]. Existing research indicates that ACLY is expressed highly in a wide range of cancers, including colorectal, liver, gastric, and prostate cancers, making ACLY a potentially effective therapeutic target for cancer by affecting lipid metabolism [102–106]. Georgia Hatzivassiliou et al. demonstrated that the knocking down of ACLY, as a key enzyme integrating glucose and lipid metabolism, limited the growth and survival of aerobic glycolytic tumor cells in vitro and reduced tumor development in vivo [107]. Given the key function in lipid metabolism, ACLY inhibitors were formerly developed for metabolic diseases. However, in recent years, ACLY inhibitors have attracted attention as promising anti-cancer drugs as more and more evidence suggests that cancer is a metabolic disease as well as a genetic one [108, 109]. For example, the ACLY inhibitor bempedoic acid (ETC-1002) was already authorized by the U.S. Food and Drug Administration (FDA) in 2020 as a non-statin LDL-C lowering drug for atherosclerotic cardiovascular disease, as well as being used in cancer treatment therapeutics [110, 111].

Taken together, enhanced PI3K/Akt/mTOR signaling induces metabolic alterations within the TME that complexly affect immune cell proliferation and growth. A multitude of different inhibitors of this pathway are in various stages of clinical trials, but only a few of them have been approved by the FDA for use in cancer therapy. In the long run, improving the targeting and sensitivity of inhibitors will further contribute to the advancement of human cancer therapy.

## LKB1-AMPK signaling pathway

**Fig. 2:**
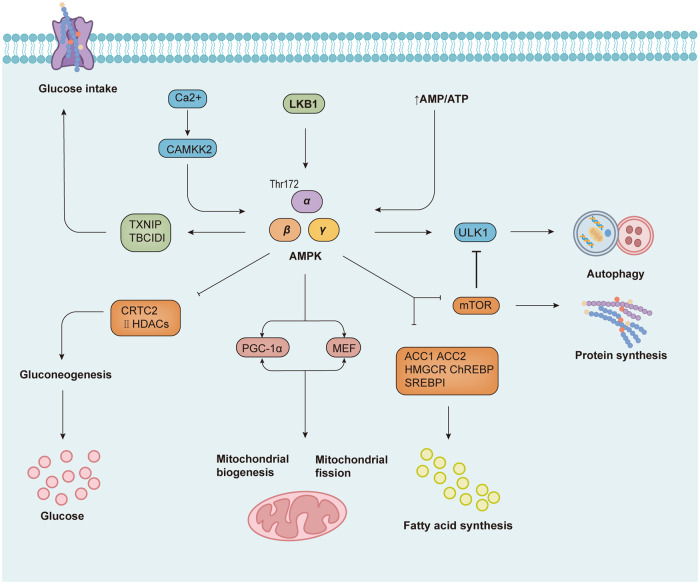
LKBI-AMPK signaling in immunometabolism. AMPK can be activated directly by LKB1 or in response to increased intracellular Ca^2+^ and lower ATP levels, acting on a range of downstream signaling molecules to inhibit fatty acid synthesis, protein synthesis, gluconeogenesis, promote glucose uptake, promote autophagy, and maintain mitochondrial function.

Another key signaling pathway linking cellular metabolism to carcinogenesis is LKB1-AMPK. LKB1 is a tumor suppressor Ser/Thr kinase that is broadly associated with cellular metabolism and proliferation, as well as modulating various cellular physiopathological processes [112]. AMPK consists of an α catalytic subunit and a β and γ regulatory subunit, which is an important hub for sensing and regulating the homeostasis of cellular energy metabolism [113–115]. When AMP binds to the γ subunit, the activation complex can be modified to make it a more susceptible substrate for phosphorylation at the threonine 172 site [116]. In addition to this, the increase in intracellular Ca^2+^ by calcium/calmodulin-dependent protein kinase kinase 2 (CAMKK2) can also activate AMPK by direct phosphorylation at the threonine 172 site [117–119]. When cells are stressed under various physiological conditions resulting in insufficient energy metabolism leading to a decrease in ATP levels, eventually AMPK will also be activated [120, 121]. LKB1-AMPK signaling exerts a core function in mediating cell metabolism, survival, and proliferation under energy stress. This is mainly manifested by the modulation of protein, lipid, and glucose metabolism in mammals, along with autophagy and mitochondrial homeostasis, which encompasses almost most of the physiological and metabolic activities of the living organism. As a key physiological energy sensor, AMPK has a series of downstream targets to exert a wide range of regulatory effects [122]. These include promoting catabolism to reduce ATP consumption and reducing anabolism to increase ATP synthesis, thereby maintaining intracellular energy homeostasis [123]. In conclusion, AMPK and mTOR interact to form a complex master metabolic network to control anabolism and catabolism and exert essential functions in the organism (Fig. 2).

As mentioned above, AMPK activation results in the downregulation of mTORC1, which conversely activates the expression of autophagy-related proteins [124]. AMPK activation also phosphorylates ULK1 and promotes its activity, activating the autophagic process [125]. In addition to linking immune signaling and cellular metabolism, LKB1 may also regulate mitochondria-related functions [126]. It has also been demonstrated that AMPK negatively regulates ferroptosis by suppressing fatty acid synthesis, as in the study by Ming-Hui Gao and colleagues, who showed that the LKB1-AMPK pathway prevented ferroptosis by inhibiting Acetyl-CoA carboxylase 1 (ACC1), the rate-limiting enzyme of fatty acid biosynthesis [127]. A collaborative study by Boyi Gan et al.‘s team also showed that AMPK could inhibit ACC, thereby reducing the formation of polyunsaturated fatty acids and ultimately suppressing ferroptosis [128]. It is thus clear that activation of AMPK is becoming a recognized therapeutic target for diseases related to metabolic disorders. On the other hand, Lei Bi and others demonstrated that suppression of the LKB1-AMPK pathway enhanced glycolysis in hepatocellular carcinoma cells, which conversely enhanced the stemness in tumor cells, thus allowing them to develop in an uncontrollable direction [129].

LKB1 has a distinct function in the differentiation and function of T cells, serving as a crucial checkpoint that collaborates with AMPK to centrally regulate lymphocyte metabolism and function [130, 131]. LKB1 acts as a critical cytokine for T-cell development and therefore contributes to the growth and survival of thymocytes. Research by Nancie J. MacIver and others confirmed that LKB1 regulated glucose and lipid metabolism in T lymphocytes, while T cells lacking LKB1 exhibited poor metabolic adaptation [132]. In conclusion, disruption of the LKB1-AMPK axis damages T-cell metabolism and over-activates mTORC1 signaling to mediate the expansion of pro-inflammatory T cells. LKB1 also coordinates metabolic resting and anti-tumor immunity of DCs. The study by Yang et al. showed that the LKB1 axis established metabolic rest in DCs to limit Treg over-expansion and Th17 cell compartmentalization, which maintains immune equilibrium or promotes an anti-tumor immune response [133]. Besides, LKB1 maintains the viability and function of Treg cells by mediating the Treg metabolism [134].

Due to the well-established phenotypic effects of AMPK activation on metabolism, AMPK is already recognized to be a promising target for the treatment of metabolic syndrome and cancer [135]. According to the site of action, AMPK activators are divided into direct activators and indirect activators. In general, direct activators directly interact with specific subunits of AMPK to activate AMPK metamorphically, such as the most widely used 5-aminoimidazole-4-carboxamide riboside (AICAR) and Thienopyridone (A-769662). Indirect AMPK activators refer to several modulators that can indirectly activate AMPK by interfering with ATP production or calcium accumulation, mostly of natural plant origin, such as metformin, curcumin, and resveratrol. Given the complex relationship between AMPK and cancer, AMPK activators currently in preclinical and clinical research focus on the treatment of obesity and diabetes, nonalcoholic fatty liver disease, and cardiovascular disease (Table 5).

**Table 5:** Potential directly and indirectly available activators of the LKB1-AMPK pathway.

Target	Drug	Phase/status	Tumor type	Treatment	ClinicalTrials. govIdentifier
LKB1-AMPK activators					
AMPK	Metformin	III Ongoing	Endometrial cancer stage I	Monotherapy	NCT04792749
		II Ongoing	Metastatic prostate cancer	Monotherapy	NCT04926155
		II Ongoing	Bladder cancer	Monotherapy	NCT03379909
		IV Ongoing	Breast cancer female	With Atorvastatin	NCT05507398
		Early I Ongoing	Prostate cancer	Monotherapy	NCT05515978
		III Ongoing	Non-small cell lung cancer	Monotherapy	NCT05445791
		I Ongoing	Advanced pancreatic cancer, advanced solid tumor	With Simvastatin, Digoxin	NCT03889795
		II Ongoing	Hepatocellular cancer, pancreatic cancer, gastric cancer, colorectal cancer	With Vitamin C	NCT04033107
		II Ongoing	Head and neck squamous cell carcinoma	With Pembrolizumab	NCT04414540
		I Ongoing	Oral cavity carcinoma	Monotherapy	NCT05536037
		II Ongoing	Preleukemia	Monotherapy	NCT04741945
		II Ongoing	Osteosarcoma Ewing sarcoma	Monotherapy	NCT04758000
		II Ongoing	Glioblastoma	With Temozolomide	NCT04945148
		I/II Ongoing	Melanoma	With Vemurafenib	NCT01638676
		I/II Ongoing	Melanoma	With Dabrafenib, Trametinib	NCT02143050
		II Ongoing	Relapsed chronic lymphocytic leukemia	Monotherapy	NCT01750567
		II Completed	Breast cancer	Monotherapy	NCT00930579
		II Completed	Prostate cancer	Monotherapy	NCT01243385
		II Completed	Colon cancer	Monotherapy	NCT03359681
		I Completed	Epithelial ovarian cancer	With Carboplatin, Paclitaxel	NCT02312661
		II Completed	Locally advanced pancreatic cancer Metastatic pancreatic cancer	With gemcitabine, erlotinib	NCT01210911
	Resveratrol	I Completed	Colon cancer	Monotherapy	NCT00256334
		I Completed	Adenocarcinoma of the colon, Adenocarcinoma of the rectum	Monotherapy	NCT00433576
	Alpha lipoic acid	I Ongoing	Head and neck squamous cell carcinoma	Monotherapy	NCT04042935

## Several immunometabolic checkpoints

Findings of immune checkpoints provide new targets in cancer therapy and have been demonstrated so in melanoma and non-small cell lung cancer [136]. However, it is still only a fraction of the patients produced significant efficacy. As researchers dug deeper into the mechanism of tumor metabolism, resistance to immune checkpoint therapy may stem from tumor cell-induced dysregulation of immune cell metabolism, which leads to immunosuppression [137]. Therefore, it may be advantageous to use metabolic pathways to kill tumor cells or reverse the metabolic vulnerability of immune cells to target cancer. Recently reported immunometabolic checkpoints with significant potential may provide new insights into anti-tumor therapies.

## IDO

IDO, known as indoleamine 2,3-dioxygenase, is responsible for the degradation of tryptophan, which can be metabolized to N-formyl-kynurenine [138]. High expression of IDO is positively associated with poor patient prognosis in various tumor types [139]. IDO promotes “metabolic, immune regulation” through catalytic oxidative catabolism of the essential amino acid tryptophan (Trp) along the kynurenine (Kyn) pathway [140, 141]. Metabolites of the Kyn pathway can exert immunosuppressive effects by acting as natural immunoreactive ligands for the aryl hydrocarbon receptor (AHR), activating Treg, and MDSCs and inhibiting immune cell functions such as effector T cells [142, 143]. Immune cells are highly dependent on Trp, and Trp depletion caused by IDO overexpression leads to an inadequate immune response [144]. Moreover, a study by Xin Zhang et al. in colitis-associated colorectal cancer showed that Treg-induced immune tolerance could be suppressed by inhibiting IDO expression and activation in tumor cells [145]. While long-standing research on IDO has focused on its ability to deplete Trp for immunosuppressive effects, Peter J Murray’s team discovered a new mechanism by which IDO promoted tumor development by transporting the IDO metabolite Kyn into cells via SLC7A11 and inhibiting ferroptosis in the tumor [146]. Up to now, IDO inhibitors (e.g., navoximod, epacadostat, linrodostat, indoximod) are being used as immunomodulators alone or in combination with other anti-tumor therapies [147, 148]. In conclusion, deeper research on existing small molecule compounds, discovering more effective IDO1 inhibitors, and improving the efficacy of combination therapy are the main research areas for IDO inhibitors.

## IL4I1

AHR is a ligand-activated transcription factor conferring flexibility to cells in sensing changes in conditions such as environment, diet, metabolites, and microbial composition [149]. AHR was initially thought to be a mediator of dioxin exerting toxicity, and as research progressed, it also proved to exert a major action in cancer and immunity [150]. As mentioned above, IDO functioned in activating AHR by depleting Trp and accumulating Kyn through the Kyn pathway. However, the difficult progress made with the combination of IDO inhibitors and immune checkpoint blockade therapies suggests that there may be other pathways of AHR activation that led to mechanisms of tolerance to IDO inhibitors in tumor cells. A study by Christiane A. Opitz and colleagues identified, through screening and analyzing a broad range of tumor cases, the highest correlation between IL4I1 and AHR activity. The results suggest that IL4I1 is a tumor-produced metabolic enzyme that mainly catabolizes tryptophan to activate AHR, enhancing tumor aggressiveness and inhibiting anti-tumor immunity [151, 152]. The available findings suggest that IL4I1 changes the anti-tumor CD8^+^ T-cell response, promotes cancer growth, affects patient survival, and may inhibit immune checkpoint inhibitor therapeutic efficacy. So, IL4I1 may be a well-established immunometabolic checkpoint [153–155].

## ACAT

ACAT is an acetyl coenzyme A cholesterol acetyltransferase that converts cholesterol to cholesteryl esters through the acetylation of cholesterol [156, 157]. In mammals, two genes encoding ACAT1 and ACAT2 were identified, and ACATs act as essential players in cellular cholesterol homeostasis [158]. It has been shown that ACAT2 was induced in some HCC tissues to establish specific cholesterol metabolic pathways for tumor cells, which in turn inhibits anti-tumor immunity [159]. Mala K. Maini et al. also found that using ACAT inhibitors to regulate cholesterol metabolism may have the unique function of directly targeting viruses and tumors, while also enhancing the clearing of viruses by T cells [160]. In addition, studies by Wei Yang and others demonstrated inhibition of ACAT1 activity increased the cholesterol levels in CD8^+^ T-cell membranes, enhanced the signaling of these killer T cells, formed more effective immune synapses, and resulted in greater sensitivity to antigens and in turn improved immune efficacy [161]. ACAT inhibitors are cholesterol-modulating drugs such as Avasimibe that are well tolerated in clinical trials as cholesterol-lowering agents, and available studies indicate that the combined use of Avasimibe and PD-1 antibodies may improve the efficacy of tumor immunotherapy even more. ACAT appears to be an attractive metabolic regulatory target, and blocking the cholesterol metabolic pathway mediated by ACAT may have a potential therapeutic effect in cancer patients.

## SIRT2

Sirtuin 2 (SIRT2) is a member of the Sirtuin family of proteins. SIRT2 is a NAD + -dependent deacetylase with proven critical functions in neurodegenerative diseases. Still, some reports of a dual paradoxical role of cancer promotion and inhibition have hindered its in-depth study [162, 163]. SIRT2 is involved in the control of cell metabolism, cell cycle, and TME [164]. Now, SIRT2 has been found to act as a key immunometabolic checkpoint for reprogramming T metabolism. Imene Hamaidi et al. found that expression of increased glycolysis and oxidative phosphorylation of T cells in SIRT2-deficient mice resulted in increased T-cell proliferation and killing capacity, which subsequently exhibited superior anti-tumor activity. The results presented that SIRT2 suppression promotes metabolic reprogramming of T cells optimally involved in aerobic glycolysis and mitochondrial respiration, maintaining T-cell effector functions in metabolically stressed TME [165]. Although the controversy over whether SIRT2 is cancer-promoting or cancer-suppressing still exists, according to some reports, SIRT2 inhibitors have shown real promise in treating cancer [166–168].

## MTHFD2

Methylenetetrahydrofolate dehydrogenase (MTHFD2) is the essential enzyme in cellular one-carbon unit metabolism, catalyzing the generation of methylenetetrahydrofolate to formyltetrahydrofolate by using NADP^+^ as the hydrogen acceptor and generating NADPH [169, 170]. Folate metabolism is a key metabolic process in organisms, providing folate intermediates to promote single-carbon metabolism, in which alterations in folate metabolism or upregulated expression of single-carbon metabolizing enzymes are thought to be involved in a higher risk of cancer [171, 172]. Overexpression of MTHFD2 in various types of cancer cells enhancing PD-L1 expression and increasing immune escape of tumor cells was studied [173, 174]. In addition, MTHFD2 expression is closely associated with mTORC1 signaling, which controls protein, lipid, and nucleotide synthesis in normal and cancer cells [175]. Recently, MTHFD2 was reported to be a metabolic checkpoint of cells that inhibits anti-inflammatory Treg cells and is a potential therapeutic target within 1 C metabolism. Mechanistically, inhibition of MTHFD2 leads to exhaustion of the purine pool, accumulation of purine biosynthetic intermediates and reduction of the trophic sensor mTORC1 signaling [176]. Therefore, in conclusion, MTHFD2 is to some extent oncogenic, which could be considered a therapeutic target and prognostic indicator for cancer, and future research directions should profoundly elucidate the mechanism of its promotion of cancer progression and accelerate the progress of functional inhibitors [177–179].

## Concluding remarks

With various immune checkpoint inhibitors (ICIs) and chimeric antigen receptor T-cell therapies led by PD-1/PD-L1 being developed, immunotherapy can be considered to have transformed the treatment of multiple cancers to some extent [180]. Still, the response rates of patients vary widely to the available immune-targeted therapies, including frequent resistance to immune checkpoint therapies [181]. A growing number of immunometabolic studies show a promising trend to increase anti-cancer effects through metabolic targets, and immunometabolism offers a broad opportunity to improve cancer therapy by modulating TME to identify new targets [182]. As the Warburg effect is well understood, reprogramming of the metabolism of the cancer cells drives the metabolic dysregulation of TME, causing partial failure of T-cell-based cancer immunotherapy. Increasing evidence suggests that the metabolic adaptations of T cells determine their function, as mentioned above, by reprogramming T-cell metabolism through checkpoints like IDO, IL4I1, and SIRT2, resulting in improved anti-cancer immune efficacy. In addition, Guo et al. found that IL-10/Fc can produce effective T-cell metabolic reprogramming through upregulation of oxidative phosphorylation, which could rejuvenate depleted T cells and augment the cancer immunotherapy response [183]. Therefore, identifying mechanisms of metabolic vulnerability of immune cells and conferring exogenous flexibility to restore the killing effect on tumor cells also seems to be a more feasible approach.

We have described the key signaling pathways of immunometabolism and some recently reported immunometabolic checkpoints, including how they affect the function and fate of immune cells through metabolic pathways, which in turn regulate the tumor immune process. On the one hand, perhaps the focus of future immunometabolic research will be to develop highly effective targeted drugs that combine specificity and safety or to improve cancer immunotherapy in combination with ICIs to improve drug resistance. On the other hand, existing immunometabolism research lacks a profound revelation of the functional and molecular regulatory mechanisms of abnormal immune cell metabolism in TME, making the clinical treatment of immunometabolism lack potential new strategies and targets.

To date, there has been growing research on the integration of the metabolic and immune domains, but there is a lack of clinical trials evaluating the metabolic interactions between immune and cancer cells assessed in human tumors. Tumor cell metabolism with heterogeneity impairs anti-tumor immunity to some extent, so clinical studies should confer metabolic advantages to immune cell populations through various pathways to better improve cancer treatment and patient prognosis. We seek to enhance the understanding of the multifaceted functions of complex immunometabolic signaling pathways in the TME and to gain a deeper understanding of immunometabolism, which we hope will benefit cancer immunotherapy.

## Data Availability

All data generated or analyzed during this study are included in this article.
